# The Physical Diagnosis of Diseases of the Lungs

**Published:** 1843-04-01

**Authors:** 


### THE PHYSICAL DIAGNOSIS OF DISEASES OF THE LUNGS. By *W. H. Walshe*, M.D. Professor of Pathological Anatomy in University College, London. Taylor and Walton. 1843.

THE object of this little book, the perusal of which will amply compensate the reader for the time spent on it, is, to present a concise view of the principles and facts of physical diagnosis as applied to diseases of the respiratory organs. It consists of Three Parts, in the first of which the various methods of physical examination, and the phenomena detected by them in health and disease, are described. The Second Part contains a tabular view of the physical causes and ordinary seat of all morbid signs in connexion with the names of the diseases, in which they occur; and also a synopsis of the signs attending each affection of the lungs, pleura and larynx; while the Third Part forms a commentary on the other two. The diseases of the respiratory organs are among those of which the physical *signs* are best understood: the *methods* employed in their detection are:—1. INSPECTION; 2. APPLICATION OF THE HAND; 3. MENSURATION; 4. PERCUSSION; 5. AUSCULTATION; 6. SUCCUSSION; 7. THE DETERMINATION OF THE SITUATION OF SURROUNDING PARTS AND ORGANS.

There being nothing of a decidedly novel character in the author's description of the mode of practising Inspection, Application of the Hand, or Mensuration, and these methods having been already noticed sufficiently by Sir J. Clarke, Stokes, Louis, and recently by Woilley, we shall merely give the morbid states ascertained by them.

### Morbid States discovered by Inspection.

A. *Form.*—The different changes of form (heteromorphism) and of position (heterotopia) of the whole chest or of its parts, which may announce subjacent disease, are referrible to the following species:—

*Expansion and Bulging.*
*Retraction and Depression.*
*Procidentia and Elevation.*
*Curvature.*
*Distortion.*

*Expansion* is a change of shape, in which one or both sides of the chest is rendered prominent; whilst *Bulging* is a local, or circumscribed expansion.

*Retraction and Depression* are the converse states of expansion and bulging.

*Procidentia* is that state in which the position of a part is slightly lower than natural; *Elevation* that in which it is higher. *Curvature* signifies that deviation of the various axes of a part in which some degree of regularity of form is retained; *Distortion*, a displacement of the same kind fundamentally, but one in which the deviations are so numerous and so considerable that all trace of regular shape is lost. The other states refer to *Size* and *Motion*.

### Morbid States discovered by Application of the Hand.

The diagnostic indications derived from *thoracic fremitus* or *vibration*, depend upon modified states of the phenomenon as produced by speaking (*vocal*) and by coughing (*tussive*), and also upon its occurrence under circumstances which do not give rise to it in health. Of the latter kind are the vibrations produced by the bubbling of air through fluids in the lung (*rhonchal*); by the collision and rubbing together of plastic matter exuded on the pleural surfaces; and lastly, by pulsation of the substance of the lung. The fluctuation of fluids contained either in the pleura or lungs is sometimes distinctly perceptible by the hand applied to the surface. The sensation is that of ordinary fluctuation, attended (not always) with a certain degree of vibratile tremor.

### Mensuration.

The object of measuring is to ascertain the comparative bulk and volume of the two sides, the relative position of their different parts, and in some few instances, the distance between those parts and certain fixed points beyond the limits of the thorax—also to ascertain the amount of expansion and retraction of the chest accompanying inspiration and expiration. Mensuration may be either general or partial:—

A. GENERAL.

| | |
|---|---|
| *a.* Circular ........ | 1. *Opposite ensiform* process.<br>2. Midway between nipples and clavicle. |
| *b.* Transverse ...... | 3. From point of one acromion to that of the other.<br>4. In axillæ.<br>5. At base of chest. |
| *c.* Antero-posterior.. | 6. Under the clavicles.<br>7. At base of chest. |
| *d.* Vertical ........ | 8. *From clavicle to most dependent point of ribs.* |

The morbid conditions to be ascertained by circular measurement are, *increase,* or *diminution of bulk* of either side as compared with the other; and defective expansion during inspiration.

B. PARTIAL MEASUREMENTS.

A. *Horizontal.—From nipple to middle line of the sternum.* B. *Vertical.* —From nipple to middle point of clavicle.

### PERCUSSION.

We now come to the next method of Physical Diagnosis, called Percussion; the immediate object of which process is the determination of the density of subjacent parts. When applied to the chest, it serves to establish any increase or diminution of the quantity of air naturally contained within that cavity. The amount of density is inferred—1st, from the nature of the *sound* elicited by percussion; 2nd, from the elasticity, or *degree of resistance,* of the body percussed. Respecting the value of changes in the elasticity, as affording diagnostic indications, our author makes some useful remarks. There are cases, he says, of not very rare occurrence in which erroneous inferences would almost inevitably be drawn from the sound elicited by percussion, were not these corrected by the information derived from the degree of resistance felt by the fingers. In illustration, he supposes the case of a cavity seated close to the surface: the unnatural clearness of sound which sometimes distinctly exists over such cavities, quite independently of an amphoric *character* in that sound, might not only lead to an incorrect estimate of the state of the subjacent part, but also to the inference that the lung in reality least affected was the most diseased. The sensation of hardness and firm resistance experienced by the fingers at once discloses the true cause of the unusual clearness.

The properties of the sound elicited by percussion, which vary with the density of the part percussed, and consequently possess practical importance, are: 1. The degree of clearness. 2. Its duration. 3. Its special character. With respect to "clearness," and its opposite "dulness," they are readily illustrated by percussing the antero-superior part of the chest and the thigh; the sounds elicited, the former *clear,* the latter

*dull*, may be used as terms of comparison for the greater number of sounds producible in various parts of the chest.

2. *Duration.*—The duration of the sound varies distinctly in different parts of the chest, for instance, at the upper part of the sternum and over the heart. The greater the dulness, the shorter is the duration of sound. With respect to the " special character " of this sound, it is to be only learned by actual trial; description cannot convey it.

With respect to the method of employing percussion, our author gives the decided preference to *mediate* percussion, the distinctive character of which is, that some solid body, interposed between the chest and percussing fingers, receives the *direct* impulse of these: in its employment two things are to be considered—the material interposed, and the agent used for striking it. The material interposed, called a pleximeter, may be of various kinds; the chief are a thin, circular or oval plate of ivory—the left index finger—or a flat piece of India-rubber. Whatever be the pleximeter used, it should be placed in close and firm contact with the surface, and, in the act of percussing, the movement should spring from the wrist only, the fore-arm and arm being held perfectly motionless. The pain occasioned to the patient in many cases, and the uncertainty of the results obtained, depend in a great measure on the non-observance of this rule. The stroke also should be *quickly* and *lightly* given, the fingers being withdrawn, or at least all pressure removed, the moment their impulse has been effectually communicated to the surface struck. To this precept there is but one exception: in eliciting a particular modification of a special character of the sound (*cracked-metal character,*) the successful production of which depends materially on the manner of striking, it is advisable to give a slow and heavy blow, and allow the fingers to press forcibly on the part for some moments after it has been given.

The position of a patient undergoing percussion should, unless circumstances prevent it, be the sitting or standing. In this precept our author agrees with Laennec, but not for precisely the same reasons. Laennec objects to the lying position from the idea of the sound being diminished by the mattress, pillows and curtains: this objection will fall to the ground, if we only recollect that our object is to obtain comparative and not direct results.

The difficulty of placing the patient perfectly level in bed, together with the constrained position in which the physician is obliged to place himself in order to get at different parts of the chest, constitute a more solid objection.

*Results of Percussion in the Natural State.*

The special character of the sound elicited from a healthy thorax can only be taught by experience.

" The duration and clearness of the sound bear a definite relation to each other; whenever the former is considerable, the latter is proportionately marked, and *vice versâ*. And again, the clearness of the sound and the sense of resistance experienced by the fingers have a manifest connexion: as the former increases, the latter decreases: with the increase of the latter, the former decreases. Thus the sound is clearer in the infra-clavicular region than in the scapular; and so the sense of resistance is much less under the clavicle, than over the scapula."

There are some exceptions, however, to this relationship of the clearness of the sound and the resistance of the parietes: these exceptions, however, only prove the rule. Thus, in the internal division of the clavicular and sternal regions, the sound is clearer than in others, for example, the infra-clavicular, where the resistance is less. This peculiarity manifestly depends on the nature of the wall of the thorax in the former situations; being there wholly composed of bone, it cannot give way and rebound under percussion to the amount which the slight density of the subjacent parts would otherwise ensure; while, on the other hand, its composition is favorable to frequency of vibration, and hence to clearness of sound.

The clearness of the sound, and, with this, its duration, together with the amount of resistance felt, varies in different parts of the healthy chest.

"A. *Anterior Regions.*—Taking the sound of the infra-clavicular regions, which is clear, and of proportional duration, as the standard, scarcely any perceptible change is perceived in examining from above downwards, till the lower part of the mammary region is reached. The change becomes still more decided in the infra-mammary regions; on the right, the sound grows considerably duller, and the resistance increases, from the presence of the liver; on the left, decrease of clearness and shortened duration are distinctly detected at the internal part of the mammary region, owing to the heart. In the internal part of the left infra-mammary region, the sound may be dull, but is more commonly clear; its special character being at the same time slightly, sometimes intensely, tympanitic, in consequence of an inflated state of the stomach. The external division of the same region, on the contrary, sometimes yields a dull sound, in consequence of the presence of the spleen. The clavicular region at the sternal extremity of the bone gives a clearer sound even than the infra-clavicular, but about the centre of the bone it becomes slightly duller, towards its humeral end considerably so. The sound obtained from the post-clavicular space is duller than that of the infra-clavicular region; even in thin persons this difference is very perceptible, though in them much less so than in fat subjects. In the upper sternal region the sound is as closely as possible the same as at the adjoining end of the clavicle. No change of consequence can be detected until we come to the inferior part of the lower sternal region; here the sound is usually dull, and may be extremely so, or, on the contrary, clear, and its special character tympanitic. The first and usual condition arises from the interference of the liver and heart; the second, from distention of the stomach with food; the third, from distention of the same viscus with gas."

After describing the variation in the clearness and duration of the sound in the different parts of the posterior and lateral regions of the chest, he next comes to the consideration of those variations in the sound of the chests of different individuals compatible with health. The sound yielded by the chest of different individuals varies in clearness; being, generally speaking, clear in proportion to the thinness of the walls. Thus it becomes distinctly clearer in persons who, from a previous state of fatness, become emaciated.

Again, the acts of inspiration and expiration modify the results of percussion in two different manners; 1, by altering the volume of the lungs; 2, by altering their density.

The following are the limits within which the pulmonary sound of percussion may be elicited. They are the same as those assigned by Dr. Williams in his work on the Chest.

" At the close of an ordinary expiration the lungs may be considered to extend on the right side as far down as the sixth rib in front, and the eighth laterally. On the left side, they reach to the seventh rib or thereabout in front, (except within two or three inches of the sternum, where they scarcely extend lower than the sixth, the heart lying in contact with the thoracic walls in that situation), and as far as the eighth rib laterally. On both sides of the chest they extend somewhat further down posteriorly than elsewhere.

" During full inspiration the lungs extend downwards in all directions somewhat further than the limits just mentioned; probably their inferior edge is then an intercostal space and a half lower than after ordinary expiration, and proportionally still lower when the lungs have been forcibly emptied of their air. At the same time the space on the left of the sternum, where, after expiration, the heart is in contact with the walls, becomes filled with lung."

Hence it is evident that the extent of surface from which the sound of percussion can be elicited, will depend on the precise moment of the respiratory act at which the examination is made.

Again, it is plain that the clearness of the sound will vary according as percussion is performed at the close of inspiration or of expiration. Hence it becomes necessary, under all circumstances, that the act of respiration be at the same stage of progress when the two sides of the chest are percussed comparatively; and the end of a full inspiration is the fittest moment for percussing.

In the state of health, change of the posture of the patient has little or no influence in modifying the relation of the lungs with respect to their containing walls; there is however one exception to this. When a person leans forward more of the heart's surface is brought in close proximity to the walls, than when he is recumbent; which will modify the limits of the pulmonary sound in the cardiac region.

### *Morbid States discovered by Percussion.*

The changes detected by percussion may be enumerated as follow :—

1. *Diminution of clearness*, gradually passing to perfect dulness, accompanied by shortened duration of sound, and increased sense of resistance.
2. Increase of clearness and of duration, with decrease of resistance.
3. Increase of clearness and of duration, with increase of resistance.
4. Alterations of special character.

With respect to the last class, or, as our author has it, the special characters, he denominates them—1. *Wooden;* 2. *Tympanitic;* 3. *Tubular;* 4. *Amphoric;* 5. *Cracked-metal.* For a description of these we beg to refer to the work itself.

When there is diminished clearness of sound, the limits within which it is detected may be *fixed* or *changeable,* the former is much the more common case, change in the posture of the patient not producing any change in the line of demarcation of the healthily and morbidly sound parts. Under certain very rare circumstances, however, the line of demarcation may be altered by making the patient change his posture; fluid in the pleura, especially if associated with air, is the only morbid state the percussion signs of which are ever thus characterised, the fluid gravitating to whatever part of the patient's chest his change of position renders the most dependent.

We now come to the subject of *Auscultation,* which may be *immediate* or *mediate;* in the former case the ear is applied directly to the chest; in the second, a hollow cylinder of wood is used as a conducting medium between the surface examined and the ear; to this the name of stethoscope has been given. Both these methods of auscultation have had their abettors and opponents. The author's opinions on the subject are very rational: he conceives that that method is to be preferred to which the observer is most habituated; and also that because cases may occur in which the ear cannot be directly applied, or in which it may be disgusting or indelicate to do so, mediate auscultation is the method with which the student should most assiduously familiarise himself; still in every instance the ear should be practised in immediate auscultation also. Among the precautions necessary to be observed in practising auscultation, he mentions that it is of importance to apply the stethoscope firmly but not forcibly to the surface, as too slight or too strong pressure interferes with the accurate transmissions, or alters the character of the sound: too slight pressure of the ear on the stethoscope is considered by some to impart somewhat of an œgophonic character to a natural resonance.

The sounds discoverable by auscultation are, A 1. The natural respiratory murmurs: 2. Certain modified conditions of these; 3. Sounds termed *rhonchi,* which occasionally supersede them; 4. Phenomena altogether adventitious, (being produced in a situation where sound is not evolved at all in the natural state of things,) but still depending on the act of respiration.

B. The resonance of the *voice.*

C. The resonance of the *cough.*

D. Phenomena common to the sounds of respiration, of the voice and of the cough.

E. The sounds of the *heart* and *vascular murmurs,* as transmitted through the tissue of the lungs.

A, 1. Each act of healthy respiration is attended by two sounds, one corresponding to the movement of inspiration, the other to that of expiration. These are the *inspiratory* and *expiratory sounds.*

The essential properties of those murmurs, those which in their modified states especially possess diagnostic value, are thus:—1. *Special Character;* 2. *Intensity;* 3. *Duration;* 4. *Liquidness;* 5. *Softness;* 6. *Rhythm.*

"By the *special character* of a sound is understood," says our author, "that essential peculiarity which must, under all circumstances of intensity, duration, rhythm, &c., distinguish it from others; the special character of the sounds of a piano-forte, for example, will invariably differ from that of the tones of a violin. Here also may be included that property of sound known as its '*quality.*' The terms *intensity* and *duration* explain themselves. The notions of *dryness* and *liquidness* of sound may be at once obtained by squeezing close to the ear first a perfectly dry, and then a moistened sponge. Similarly, if we press together a mass of wool held beside the ear, the property of *softness* in sound will at once become intelligible; its converse, *hardness,* by grating together any two hard bodies. The *rhythm* of a sound means its mode of progression or evolution, which may be continuous and equable, or interrupted and jerking."

Our author cautions the student against confounding this signification of the term *rhythm,* as applied to a *single* sound, with its more common meaning in medical language when referring to the regularity and mode of succession of two or more distinct and separate sounds, as for example, of the two respiratory murmurs.

Every sound detected by auscultation should be analysed with respect to these various properties.

There is a difference in the nature and properties of the murmurs, depending on the various divisions of the respiratory organs from which the sounds audible externally are transmitted: in this respect they are called, A, *pulmonary* or *vesicular;* B, *bronchial;* C, *tracheal;* D, *laryngeal;* E, *pharyngeal.* These agree in this particular—that in all of them the audible sound may be resolved into two—an inspiratory and expiratory.

A. *Pulmonary or Vesicular Respiration, (as originally employed by Laennec.)*

The term pulmonary has been replaced by the term *vesicular,* which was employed originally to designate the *seat* of these sounds; some however have incorrectly referred the term vesicular to the *character* of the sound. There is nothing however, as our author observes, in the nature of the respiratory murmurs suggestive of a connexion with vesicles, and whenever such character occurs the phenomenon it attends is morbid.

"The *inspiratory* murmur is a sound of gentle breezy character, neither liquid nor dry; soft, of a certain intensity and duration, and in respect of rhythm, gradually developed and continuous.

"The *expiratory* murmur possesses identically the same properties as the inspiratory, except in respect of intensity and duration. It is about three or four times less intense and shorter than the latter sound."

We recollect that Fournet expresses the ratio of the inspiratory to the expiratory murmurs by 5·1, in respect of intensity and duration; which ratio that gentleman considers to be the same in all subjects. Our author disagrees with M. Fournet in that particular; he considers that the excess of intensity and of duration is always on the side of inspiration, and this to a considerable amount.

The continuousness of the inspiratory and expiratory murmur, it should be observed, is an important character of the *healthy* pulmonary respiration.

There are certain variations of the respiratory murmurs which may exist within certain limits in the state of health; that is, there are healthy varieties of respiration. These may be referred to age; the part of the chest examined; the rapidity and fulness of respiration; temperament and idiosyncracy.

After considering the *natural* or *healthy* murmurs in the different sections of the respiratory organs, our author now comes to describe them in their *unnatural* or *unhealthy* states.

A. 2. *Modified Conditions of the Respiratory Murmurs.*

*Pulmonary* or *Vesicular.* It is very rare to find one only of the primary properties of the respiratory sounds affected; generally speaking, two or more of them suffer alteration at the same time; and thus are produced compound conditions of change, or distinct species, which may be classed as follow:—

Species of unhealthy respiration, distinguished by changes of

| | | |
|---|---|---|
| 1. *Duration and Intensity.* | *a.* Exaggerated respiration.<br>*b.* Weak respiration.<br>*c.* Suppressed respiration. | |
| 2. *Rhythm* solely, or with other properties. | *d.* Incomplete respiration.<br>*e.* Jerking.<br>*f.* Divided. | |
| 3. *Character,* with other properties. | *g.* Harsh respiration.<br>*h.* Bronchial.<br>*i.* Blowing. | 1. Diffused.<br>2. Tubular.<br>3. Cavernous.<br>4. Amphoric. |

The *jerking respiration* is thus explained. "When the movement of inspiration, instead of being accompanied by a murmur continuous from the outset to the close (which may be thus represented ∿∿∿∿∿∿∿∿∿∿) is attended with a sound of an interrupted character, divided into several unequal parts (thus ∿∿∿ | ∿∿ | ∿∿∿ | ∿∿∿ &c.) the respiration may be called *jerking*. The expiratory sound does not possess this peculiarity, but is generally somewhat increased in duration, while the inspiratory is certainly somewhat decreased in this respect." f. *Divided respiration* is said to exist when the inspiratory and expiratory murmurs, instead of succeeding each other so closely, as to be in a manner continuous, are separated by a distinct interval.

A, 3. *Sounds superseding the Respiratory Murmurs: Rhonchi.*

"*Rhonchus* is an unnatural sound audible in inspiration or in expiration, or in both; of dry or humid character; masking the natural murmurs more or less completely; persistent or interrupted; originating in the minute or the larger bronchi, in excavations of the pulmonary substance, or (probably) in the tissue of the lung itself; and produced either by the passage of air along bronchi of altered caliber, by air bubbling through fluid contained in those tubes, or (probably) in certain conditions of the pulmonary substance by motions of that substance on itself."

With respect to the seat of the rhonchus being in the tissue of the lung itself, our author here alludes to the crepitant rhonchus, the mechanism of which is still undecided. Two points require investigation with respect to this matter: *a.* The intimate seat of production of the rhonchus; *b.* The physical condition of that seat at the moment of production. According to some, the cavities of the pulmonary cells are the cause of the rhonchus; because (says Andral) the rhonchus is evidently a diminution of the finer mucous bubbling rhonchus, which is confessedly produced in the smaller bronchi. This is an obvious begging of the question, as our author well observes, the difficulty being to ascertain whether the rhonchus is a diminutive of the mucous. The same view of the seat of this rhonchus is entertained by other writers, and supported by plausible, though certainly not unanswerable, arguments.

The following arrangement of rhonchi, founded on their amount of liquidness and their special character, appears to our author the most practically useful.

| *Degree of Liquidness.* | | | *Special Character.* |
|---|---|---|---|
| A. *Dry.* | *a.* Of shrill tone. | 1. Sibilant. | Whistling.<br>Clicking. |
| | *b.* Of grave tone. | 2. Sonorous. | Snoring.<br>Rubbing.<br>Cooing. |
| | *c.* Of tone rather shrill than grave. | 3. Dry crackling. | |
| B. *Humid.* | 4. Crepitant. | | *a.* Primitive.<br>*b.* Redux. |
| | 5. Subcrepitant. | | *a.* True Subcrepitant.<br>*b.* Humid.<br>*c.* Continuous. |
| | 6. Humid Crackling. | | |
| | 7. Mucous, Submucous. | | |
| | 8. Cavernous, Cavernulous. | | |
| *Of Doubtful Nature.* | "Pulmonary crumpling sound."<br>Dry crepitant with large bubbles. | | |

For a minute account of these varieties we must refer to the author himself.

A. *Phenomena altogether adventitious, being produced in a situation where sound is not evolved at all in the natural state of things.*

The phenomena alluded to here are the various friction-sounds produced by the movement of the opposite laminæ of the pleuræ on each other in a state of disease.

The modifications of intensity and special character of friction-sound justify the establishment of four varieties: *a*, the *grazing; b, rubbing; c, grating; d, creaking.*

### *Modified Conditions of the Respiratory Murmurs in the Trachea and Larynx.*

These serve as useful auxiliaries in the diagnosis of many cases of laryngeal disease, especially when compared with the results of bronchial and pulmonary auscultation; without such comparison it is very difficult sometimes to say whether a given rhonchus is actually produced in the larynx or transmitted from the bronchi.

The chief morbid phenomena occurring in the larynx are—

Harsh respiration.

Sibilant, Sonorous, Valvular, Gurgling, Flapping } Rhonchi.

B. *The Resonance of the Voice.*—In auscultation of the voice it is important that its intensity and tone be precisely the same while both sides of the chest are examined; and in order to ensure this uniformity, the observer may cause the patient to count a certain number of figures at a measured and even rate. In general a loud tone should at the same time be directed. The stethoscope should be laid firmly upon the surface, and the ear applied to the instrument, but without any degree of forcible pressure; if either be too lightly applied, a tremulous bleating character may be given to the resonance, if too forcibly, the distinctness of this is diminished.

The condition known as exaggerated resonance, or diffused bronchophony is probably more accurately appreciable by means of immediate than mediate auscultation: all other unnatural states of vocal resonance are more satisfactorily and distinctly ascertained with the stethoscope.

The amount and special character of the natural resonance are modified by certain circumstances altogether independent of disease. The chief of these, and the modes of modification they produce, are as follow:—

1. The natural resonance is *cæteris paribus* marked in proportion to the graveness of the voice. This statement is only true of intensity, however; there is no greater tendency to concentration or articulation of the sound when the voice is grave than when it is shrill.

2. The natural resonance of the voice is, as a corollary from the last proposition, more marked in males and in adults than in females and in children.

3. The special character of the resonance varies with the quality of the speaking voice.

4. The natural resonance is more strongly developed the larger the chest, and the less loaded its walls with fat and muscle.

5. It is stronger in front than behind (with the exception of the interscapular region) and at the upper than the lower parts of the thorax.

6. It is equal on both sides of the chest, except under the clavicles, and in the spaces between the spines of the scapulæ and median line: in these regions the phenomenon is more strongly marked on the right side.

It may be well to observe here, that it is M. Louis's opinion, with which our author coincides, that in the space corresponding posteriorly to the origin of the right bronchus, the voice resounds more strongly than in the same part on the left side. The natural resonance also is more intense under the right than the left clavicle. The application of this fact to the diagnosis of early tuberculisation is of considerable value.

*Unnatural or Morbid Vocal Resonance.*—The modifications of natural resonance which arise in disease may be classed as follow:—

*Vocal Resonance.*

| | |
|---|---|
| Diminished in intensity. | 1. Weak resonance.<br>2. Suppressed resonance. |
| Increased in intensity. | 3. Exaggerated resonance.<br>4. Bronchophony. |
| Increased in intensity and altered in special character. | 5. Œgophony,<br>6. Pectoriloquy.<br>7. Amphoric resonance. |

Laennec's varieties of pectoriloquy, viz. perfect, imperfect and doubtful, our author considers inadmissible, and they are referrible to bronchophony. He conceives his error to have arisen from assuming that pectoriloquy must always exist when excavation is present. So that whatever resonance he met in connexion with a cavity, he styled pectoriloquy.

In certain cases pectoriloquy has a tremulous, and so acquires some slight degree of œgophonic, character. This is occasioned, according to MM. Barth and Roger, when the cavity affording it, has a flattened form, and is provided with walls capable of moving backwards and forwards under the influence of vibration.

*Tussive Resonance.*—The cough of healthy subjects, when ausculted on the surface of the chest, furnishes a quick, short, commonly dull and indistinct, somewhat diffused sound, without hollow or tubular character, but attended with a distinct sensation of succussion of the interior of the thorax.

The *modified states* of the pulmonary cough, which occur in disease, are the *Bronchial, Cavernous* and *Amphoric*.

*Metallic Tinkling.*—This phenomenon, observes our author, occurring in connexion with respiration, co-exists commonly with inspiration, being prolonged somewhat into the expiration following, and is very rarely limited to the latter. M. Fournet, however, places it among the phenomena co-existing with both inspiration and expiration, but especially with expiration.

*Sounds of the Heart and Vascular Murmurs transmitted through the Substance of the Lungs.*

In subjects having all their thoracic organs healthy, and those affected with *cardiac* (without pulmonary) disease, the intensity of the heart's sounds will be directly as the distance of the point at which they are examined from their centre of production. On the other hand, in cases where the heart is healthy, but the lungs diseased, this regular order of propagation is liable to alter, in consequence of change in the conducting power of the media intervening between that organ and the surface where auscultation is performed. The positive intensity of sound produced in the heart is unaltered, but its relative intensity, as discovered at different parts of the thoracic surface, is changed. Upon this fact are founded indirectly some inferences bearing upon the diagnosis of affections of the lungs and pleura. This source of diagnosis has as yet been applied in the investigation of but few diseases: the signs furnished by it are—*increased*

*intensity of transmission of heart's sounds.* Diminished intensity of transmission of heart's sounds.

### DETERMINATION OF THE SITUATION OF SURROUNDING PARTS AND ORGANS.

The object of attempting to determine the situation of other parts than the lungs themselves, when the diseases of these organs are the subject of investigation, is, as might be anticipated, to infer from any change in that situation, the existence of some pulmonary affection capable of producing it. The organs and parts liable to undergo displacement in consequence of pulmonary disease are—1. The heart; 2. The mediastinum; 3. The diaphragm; 4. The liver; 5. The spleen; 6. The stomach.

The existence of displacement of these organs is determined by inspection, by application of the hand, by percussion, and by auscultation.

The modes of displacement from its natural situation are—

*a.* Lateral detrusion { to the right side. / to the left side. }

*b.* Elevation.

*c.* Procidentia.

Part II. of this work consists of a Table exhibiting the physical cause and ordinary seat of the different physical signs, together with the names of the diseases in which they are observed. We shall extract a few examples by way of illustrating the nature and uses of this excellent Table.

### SIGNS DISCOVERED BY INSPECTION.

| *Name of Sign.* | *Physical Cause of Sign.* | *Ordinary Seat of the Sign.* | *Diseases in which it is observed.* |
|---|---|---|---|
| EXPANSION. | Gradual and general detrusion of the walls of the chest, by a force acting from within outwards—the elasticity of the lung having been first destroyed. | The left side of the thorax; because its most usual cause (pleuritic effusion) is most common on that side. | Pleuritic effusion.<br>Pleuro-pneumonia.<br>Hydrothorax (rare)<br>Cancer of the pleura or lung.<br>Hæmothorax.<br>Pueumonia. |
| BULGING. | The same cause acting locally. | At either base: oftenest the left.<br>Infra-clavicular region.<br>Post-clavicular.<br>Anterior surface, generally.<br>Mammary and central sternal region. | Gravitating pleuritic effusion.<br>Pleuro-pneumonia.<br>Cancer of the pleura or lung.<br>Hypertrophous emphysema. |

### Signs discovered by Application of Hand.

| *Name of Sign.* | *Physical Cause of the Sign.* | *Ordinary Seat of the Sign.* | *Diseases in which it is observed.* |
|---|---|---|---|
| Increased vocal and tussive vibration. | Unnatural density of the pulmonary substance between the bronchi and the part examined. | Infra-clavicular and latero-inferior regions. | Tubercles. Pneumonia. Dilatation of bronchi. Chronic consolidation of the lung. Pulmonary apoplexy. Pulmonary œdema. |
| Rubbing vibration. | Walls of chest set in vibration by friction of pleural false membrane. | Latero-inferior part of chest. | Pleurisy, periods of { Plastic exudation. Absorption, with or without Retraction. |

### Signs discovered by Mensuration. (circular.)

| *Name of Sign.* | *Physical Cause of Sign.* | *Ordinary Seat of Sign.* | *Diseases in which it is observed.* |
|---|---|---|---|
| Increased bulk of either side. | Gradual detrusion of the walls of the chest by a force acting from within outwards, the elasticity of the lung being first destroyed by pressure. | The left side of the thorax. | Pleurisy, (period of effusion with dilatation.) Hydropneumothorax. Pneumo-thorax. Cancerous tumor of the lung or pleura. |
| Diminished bulk of either side. | Long continued pressure having reduced the lung to a very small size, the pleuritic fluid is removed by absorption; the lung being unable to recover its previous bulk, the chest yields inwards under atmospheric pressure. | The left side of the thorax. | Pleurisy, period of absorption with retraction. |

We shall now take a few extracts from that division of the Second Part of this work which constitutes a SYNOPSIS OF THE PHYSICAL SIGNS OF DISEASES OF THE LUNGS.

### ACUTE PNEUMONIA.

### FIRST STAGE—ENGORGEMENT.

*Inspection.*—Diminution of motions of expansion and elevation (if severe pain be present.)

*Percussion.—Sound less clear than natural, resistance slightly increased.*

*Auscultation.—Respiratory murmurs weak, suppressed or masked by rhonchus in the affected parts; exaggerated in those at some distance from it and in the opposite lung; true crepitant rhonchus; vocal resonance somewhat encreased;* some degree of bronchial cough.

### SECOND STAGE.—RED HEPATIZATION.

*Inspection.*—Expansion of the affected side. Bulging of the infra-clavicular sub-region in pneumonia of the upper lobe; diminution of the motions of expansion and elevation; motion of expansion diminished in proportion to that of elevation.

*Application of the Hand.—Increased vocal and tussive vibration;* pulsatile vibration.

*Mensuration.*—Increase in the semicircular measurement of the side; deficient increase in semicircular width in inspiration.

*Percussion.—Sound diminished in clearness, until completely dull, decreased in duration, sense of resistance very much increased;* under certain circumstances of locality of the inflammation character of the sound tubular.

*Auscultation.—Respiration bronchial, or blowing, of either the diffused or tubular varieties;* weak in the immediate vicinity of the inflamed part; *exaggerated in more distant parts, and in opposite lung; bronchophony,* or (under certain circumstances) *broncho-œgophony;* bronchial cough; intensity of transmission of heart's sounds increased.

### THIRD STAGE.—GREY HEPATIZATION, OR INTERSTITIAL SUPPURATION.

The signs the same as the preceding one; facts observed of late years tend to render it probable that the occurrence of a peculiar form of mucous rhonchus, in addition to the signs of the second, may announce the supervention of the third stage.

### STAGE OF RESOLUTION.

*Inspection.*—Retraction or depression of the affected side.

*Mensuration.*—Diminution of semicircular width.

*Percussion.—Dulness of sound less marked than previously, and gradually decreasing.*

*Auscultation.—Respiratory murmurs weak and harsh; redux crepitant,* or sub-crepitant rhonchus; still some bronchophony, gradually disappearing.

### PLEURISY.

### *a.* DRY PERIOD.

*Inspection.—Diminished motions of expansion and elevation; jerking rhythm of these motions,* partial motions also slightly lessened in amount.

*Percussion.—Clearness of sound not perceptibly diminished.*

*Auscultation.—Intermittent weak respiration; occasionally, but rarely, grazing variety of friction-sound.*

### b. PERIOD OF PLASTIC EXUDATION.

*Inspection.—Signs same as before.*

*Application of the Hand.—Rubbing vibration occasionally to be felt.*

*Percussion.—Clearness and duration of sound somewhat diminished;* if notably so, and the sensation of resistance very slightly but distinctly increased, the plastic matter is abundant; *deep respiration will restore, in a great degree, the natural clearness of sound.*

*Auscultation.—Intermittent weak respiration; rubbing or even grating variety of friction-sound.*

### c. PERIOD OF EFFUSION.

#### c, 1. *Of Laminar Effusion.*

*Inspection.—Signs usually as in the previous periods, but sometimes the partial and general motions become freer, and cease to be jerking in consequence of decrease of pain.*

*Application of the Hand.—Diminution of vocal and tussive vibration, rubbing vibration, if before perceptible, ceases to be felt.*

*Percussion.—Sound diminished in clearness and in duration; sense of resistance increased: these changes exist to an equal amount all over the chest, and are not influenced by any change of posture of the patient.*

*Auscultation.—Deep-seated persistent weak respiration,* with harsh or slight bronchial character; *friction-sound ceases commonly to be audible; vocal resonance louder than natural, and generally having some œgophonic character,—this unnatural resonance being diffused, though commonly most marked towards the angle of the scapula.*

#### c, 2. *Of Gravitating Effusion.*

*Inspection.*—Motions of expansion and elevation, and costal motions much diminished, especially at the lower parts of the chest.

*Application of the Hand.—Vocal and tussive vibration abolished at the inferior parts of the chest*; rubbing vibration not perceptible.

*Mensuration.*—Defective expansion of the chest in inspiration.

*Percussion.—The upper part of the chest is found to have recovered, in some degree, its natural sound: the sound of the lower is completely dull and proportionally short, the sense of resistance here extremely marked; the limits of the dull and clearer-sounding parts are distinguished by a tolerably well-defined line; the limits of the dull sound commonly change with the position of the patient;* deep inspiration has no influence on the limits or degree of the dull sound.

*Auscultation.—Respiratory murmur suppressed where effusion most abundant; weak where less so;* in some comparatively rare cases, however, *the respiration is distinctly audible, and of the diffused blowing type in the parts directly corresponding to the effusion; above the effusion they are exaggerated, harsh, or bronchial, friction-sound almost always inaudible, sometimes, however, may be slightly detected towards the upper edge of the effusion, where also œgophony* is heard, especially towards the angle of the scapula; *œgophony may be absent or replaced by bronchophony.*

#### c, 3. *Effusion with Dilatation and Detrusion.*

*Inspection.—Affected side expanded; intercostal spaces widened, flat or even convex; motions of expansion almost completely abolished;* lower part of chest slowly dragged upwards, a motion which seems to take place later than on the other side; *costal motions abolished;* fluctuation visible in rare cases of considerable bulging of the intercostal spaces.

*Application of the Hand.*—Surface felt to be unnaturally smooth and even; *vocal and tussive vibration not to be detected*; simple fluctuation producible in cases of bulging of the intercostal spaces; peripheric fluctuation.

*Mensuration.—Increase of semicircular measurement of the side; deficient enlargement of side during inspiration; antero-posterior diameter increased; vertical measurement also increased;* distance between the nipple and median line greater than on the opposite side.

*Percussion.—Sound completely dull, and of short duration where the fluid exists; resistance extremely marked;* the limits of the dull sound not altered by changing the position of the patient.

*Auscultation.—Respiratory murmurs totally suppressed, except close to the spine and under the clavicle, here harsh, bronchial, or even slightly blowing, sometimes more extensively audible of the latter type;* friction-sound inaudible: œgophony or other vocal resonance ceases commonly to be perceptible.

*Situation of Surrounding Parts.—Heart and mediastinum detruded to the opposite side; this corresponding division of the diaphragm depressed with the subjacent abdominal viscera.*

### *d.* PERIOD OF ABSORPTION.

#### d, 1. *Without Retraction of the Chest.*

*Inspection.—The appearances of enlargement and bulging gradually disappear, and with them the obstracted state of the general and partial motions;* fluctuation ceases to be visible.

*Application of the Hand.—Natural intercostal depressions again felt,* increased by emaciation; rubbing vibration sometimes re-appears, as also *vocal and tussive vibration.*

*Mensuration.—The semicircular and vertical measurements fall to the natural standards;* the distance between the nipple and median line decreases gradually to the natural amount.

*Percussion.—Sound gradually recovers its natural clearness and duration, first at the upper then at the lower parts, at the latter* it may *long retain some degree of dulness; the sensation of resistance alters in the same way.*

*Auscultation.—Respiratory murmurs gradually restored, but* remains for a variable time weak *and slightly harsh; friction-sounds sometimes re-appear and continue audible for an indefinite period; œgophony or bronchophony (redux) re-appear.*

*Situation of the Surrounding Parts.—Heart, mediastinum, vault of the diaphragm, and subjacent abdominal viscera restored to their natural position.*

#### d, 2. *With Retraction of the Chest.*

*Inspection.—Retraction, or more commonly depression; procidentia of the shoulder, of the ribs, and of the nipple; scapula tilted outwards, at its inferior angle;* distortion of the ribs; *intercostal spaces unnaturally narrow; diminished motions of expansion and of elevation, especially of the former; while the latter is affected in the same manner as during the period of effusion with dilatation; motions of ribs on each other much impaired.*

*Mensuration.—Semicircular measurement diminished; antero-posterior diameter diminished; distance between the nipple and the middle line diminished.*

*Percussion.—Sound dull and of short duration, with marked resistance under the finger at lower parts; superiorly it is clearer, in the inferior regions it has a wooden character, and, at the antero-superior, often a tubular one.*

*Auscultation.—Respiratory murmurs suppressed at base, at upper parts weak and harsh, or bronchial; friction-sounds commonly audible, of rubbing, grating, or creeking type; bronchophony and bronchial cough, especially posteriorly.*

*Situation of Surrounding Parts.—The vault of the diaphragm and subjacent viscera are sometimes drawn above their natural level; mediastinum and heart commonly, but not always, restored to their natural positions.*

We here close our analysis of this valuable little manual, not, however, without recommending it earnestly to the careful perusal of every medical student, who would wish to make himself acquainted with all the modern improvements in thoracic semeiology.

